# Nonsurgical Management of a Tooth With Intracanal Fiber Post and Periapical Lesion Using Guided Endodontic Technique

**DOI:** 10.1002/ccr3.71240

**Published:** 2025-10-14

**Authors:** Mamak Adel, Zohreh Asgari

**Affiliations:** ^1^ Department of Endodontics, Dental Caries Prevention Research Center Qazvin University of Medical Sciences Qazvin Iran; ^2^ Department of Endodontics, School of Dentistry Qazvin University of Medical Sciences Qazvin Iran

**Keywords:** fiber post, guided endodontic, periapical lesion, post removal

## Abstract

Several techniques have been proposed to remove fiber posts that entail risks. Guided endodontics can enable safe and minimally invasive fiber post removal in teeth requiring nonsurgical retreatment, improving prognosis and preserving tooth structure.

## 1 Introduction

While a post is present on a tooth with previous endodontic treatment and a periapical lesion develops, the post needs to be removed for nonsurgical root canal treatment to succeed. In some cases, removing the post is necessary to improve a new restoration's esthetics and mechanical properties [1]. Adhesively bonded glass, carbon, or quartz fiber posts have gained popularity in recent years, substituting for metal posts [2, 3, 4]. Fiber posts are bonded inside the root canal using adhesives such as glass ionomers or composite resins, which are frequently difficult to remove [5]. Different methods for removing a fiber post have been suggested, including using a round bur, an ultrasonic tip, and a specialized post removal kit. The studies show that these techniques entail risks [6, 7, 8]. These risks include the increased frequency of root perforation, axis deviation, and root fracture, which result in a lower tooth survival rate [9]. Additionally, these methods have high chairside time and require a clinician's experience [10, 11, 12].

Guided endodontic procedures were proposed for the nonsurgical root canal treatment of teeth with pulp canal obliteration. These methods have achieved favorable results due to good clinical outcomes, particularly for managing calcified canals [13, 14, 15]. Despite the recent introduction of their use for fiber post removal, more research is required for their clinical application. The technique suggests considerable advantages, whereas extensive research is conducted in laboratory studies, with inadequate clinical data to evaluate its effectiveness [16].

In order to create a treatment guide via 3D printing, the guided endodontic technique combines the three‐dimensional data obtained from cone‐beam computed tomography (CBCT) devices with the surface data of teeth collected by an intraoral scanner [17, 18]. The drill can access the root canal by adjusting the guide to fit the tooth's surface [19]. This innovative technique offers more accuracy to prevent nonessential removal of healthy tooth structure and subsequent problems. Also, guided endodontic improves the treatment prognosis and predictability of outcomes, resulting in enhanced patient satisfaction [20, 21]. This case report demonstrates post removal from an infected maxillary central incisor safely and efficiently employing a guided endodontic technique.

## 2 Case History/Examination

**FIGURE 1:**
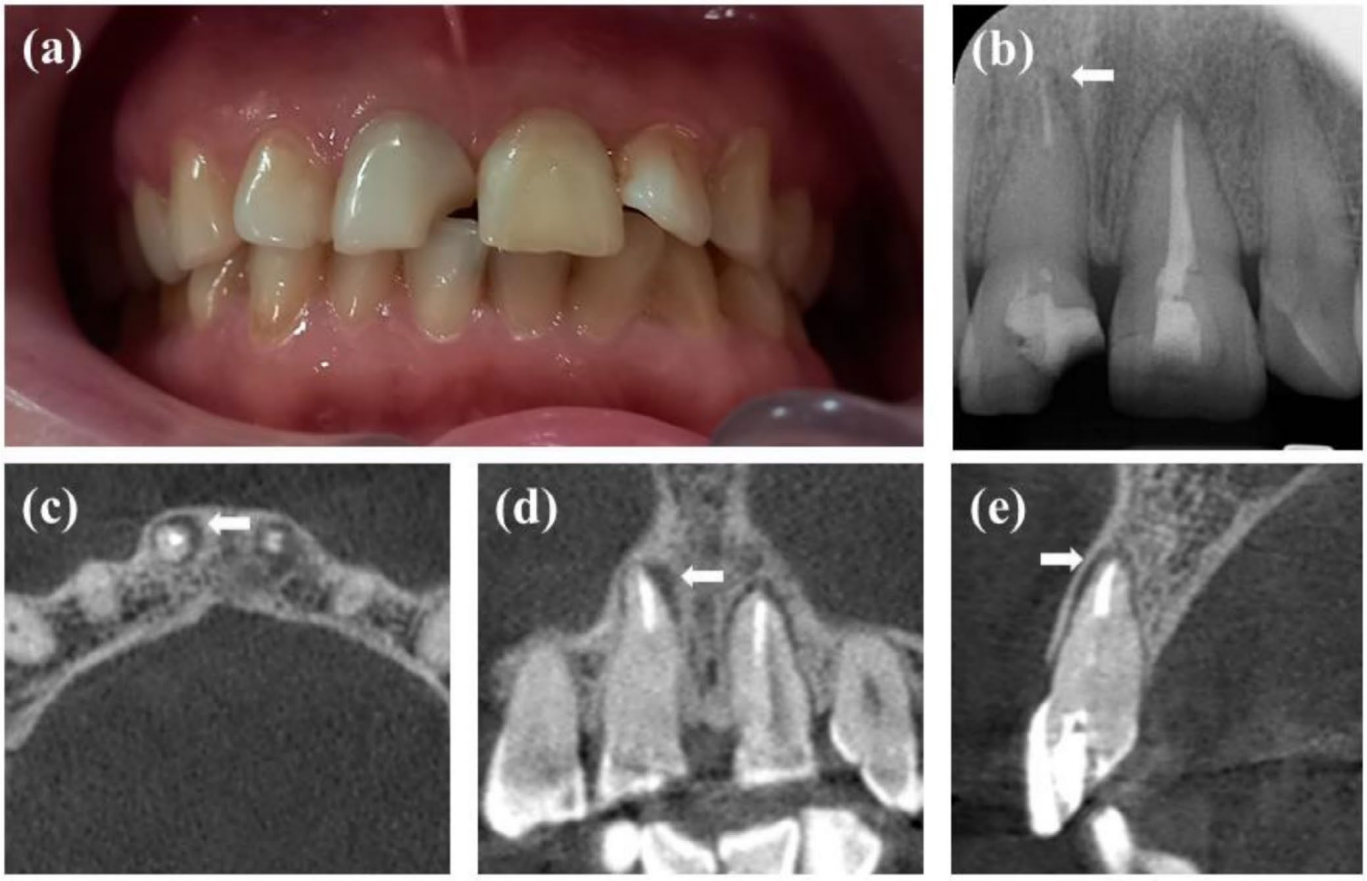
(a) Perioperative clinical photography showing a fractured tooth #8. (b) Preoperative radiography showing preinstalled fiber post and periapical lesion. (c–e) Preoperative CBCT assessment. Axial, coronal, and sagittal planes confirm a radiolucent area with intact buccal and palatal plates.

A 44‐year‐old normal healthy female patient, ASA1, was referred to the postgraduate endodontic clinic at the School of Dentistry, Qazvin University of Medical Science, to treat a fractured maxillary right central incisor. Based on the declaring patient, the affected and contralateral teeth underwent endodontic treatment 5 years ago and a month ago. In the clinical examination, tooth #8 exhibited mild sensitivity to the percussion test, while the mobility and periodontal probing depth were within normal limits (Figure 1a). A periapical radiograph showed a previous endodontic treatment and an associated periapical lesion. It had been restored using a fiber post (Figure 1b). To obtain identification of the root anatomy, intracanal fiber post position, possible fracture of the root, and extent and location of the periapical lesion, a CBCT of the maxillary arch was performed using the Promax 3D (Planmeca, Helsinki, Finland), operating at 110 kV, 11.21 mA, with a FOV of 8 × 8 cm, and a voxel size of 0.15 mm. A periapical radiolucency with intact buccal and palatal plates was shown on the imaging (Figure 1c–e).

## 3 Diagnosis and Treatment

The tooth was diagnosed as previously endodontically treated and with chronic apical periodontitis. Due to the requirement of intracanal retention, removing the cemented fiber post was necessary. During fiber post removal, minimizing tooth structural loss was essential for this compromised tooth. Thus, we chose to treat this tooth using the guided endodontic technique. Consent was acquired after the patient was informed of the procedure's risks and advantages.

**FIGURE 2:**
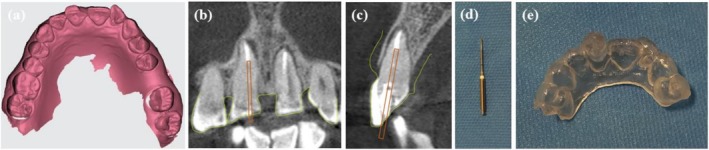
(a) Intraoral scan of the maxillary arch using STL format. (b, c) Virtual planning of coronal and sagittal plane of the guide drill placement to remove the fiber post. The drill was positioned along the long axis of the fiber post set up to its apical tip. (d) Munce discovery bur #2. (e) Endodontic guide. The STL file and the DICOM file obtained by CBCT were virtually merged, and a guide was fabricated using a 3D printer.

An intraoral scan was obtained from the upper arch via the CS 3600 scanner to fabricate a customized stent (Carestream Health, Atlanta, Georgia, USA) (Figure 2a). Both the 3D data sets, including optical scanning data (Standard Tessellation Language‐STL) and CBCT data (DICOM file), were imported into the BlueSky Plan software (Blue Sky Bio). The software automatically combined the two mentioned files. The software designed a virtual path of the guide drill based on the diameter, location, and apical extent of the fiber post seated in the tooth. Moreover, a guide stent was designed (Figure 2b,c). To assist in guiding the drill, a guidance hole was made, and its dimensions were taken. This procedure was carried out using Munce discovery bur #2 (Meisinger, Germany), which measured 16 mm in length and 1 mm in diameter (Figure 2d). To achieve maximum stability, the template was extended to cross the middle. The virtually designed guide with a thickness of 3 mm and an offset of 2 mm was fabricated using a 3D printer (BIO3D, Bio3D Ltd., Seoul, Korea) (Figure 2e).

**FIGURE 3:**
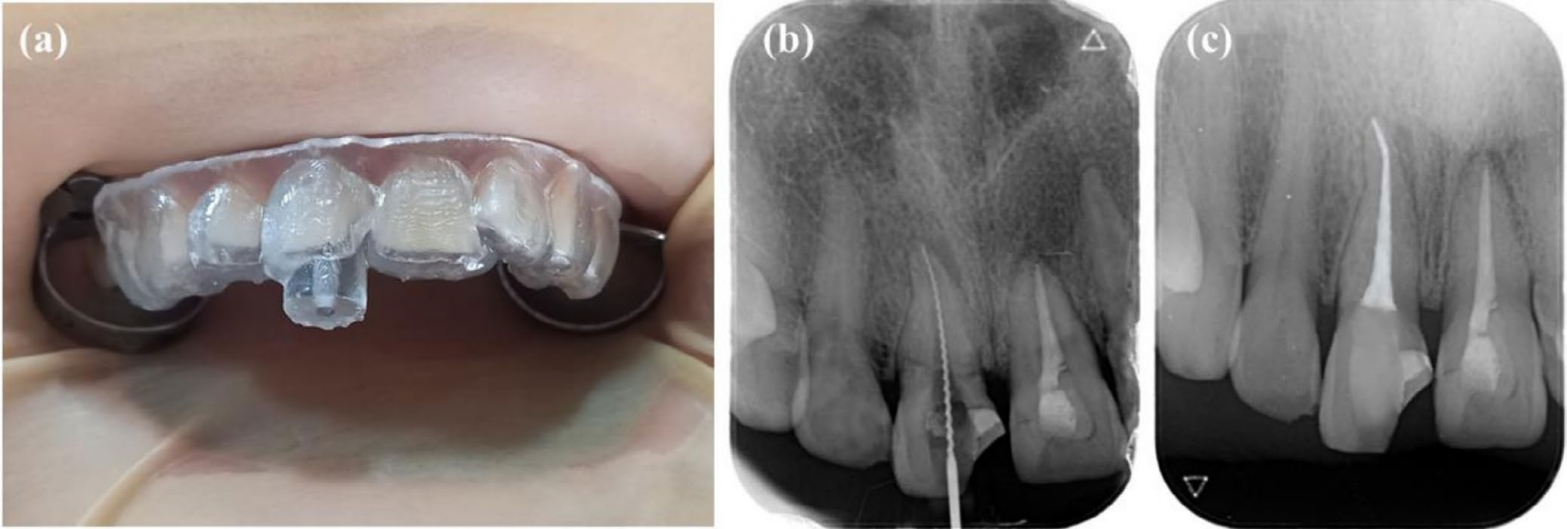
(a) Under rubber dam isolation, the guide was positioned on the teeth to check the correct fitting. (b) Working length determination radiograph. (c) Postoperative radiograph with temporary filling.

In the treatment visit, the stent was initially placed in the patient's mouth to ensure it would fit perfectly. The accurate fit of the stent was checked by taking a wash impression (Zhermack, light body, Italy). A rubber dam was placed around 10 teeth after local anesthesia (2% lidocaine containing 1:80,000 epinephrine). The precise fitting of the stent was confirmed again (Figure 3a). Subsequently, drilling was performed under an operative microscope (MediWorks SM620, China) using a guide drill (Munce discovery bur, #2) with a rubber stopper used at 40,000 rpm under irrigation, via up‐and‐down movement through the stent's guidance hole. This process was performed intermittently in a three‐step, until the gutta‐percha was exposed. At each step, the stent was taken off, the root canal irrigated with normal saline, the path was verified with a K‐file #15 (Mani Inc., Tochigi, Japan) under magnification, and the drill was cleaned. This approach made it possible to clear debris completely and for the drilling axis to be evaluated by an operative microscope. After the drilling completion and periapical radiograph, the remaining fiber post‐resin core was eliminated using an ultrasonic tip (Start‐X #3, Dentsply, Switzerland). After the coronal portion of the gutta‐percha was revealed under magnification, the remaining gutta‐percha was removed employing a size 25 H‐file (Mani Inc., Tochigi, Japan) and chloroform (Golchadent, Iran). Working length was determined with a size 25 H‐file using an electronic apex locator (Woodpex V, Woodpecker, China) and confirmed by radiography (Figure 3b). Following, ProTaper Gold system rotary file instruments (Dentsply, Sirona, USA) # F1–F3 and 5.25% NaOCl irrigation were used for complete shaping and cleaning, respectively. The final irrigation was carried out by a sonic device (EndoActivator, Woodpecker, China), which began with 5.25% NaOCl and proceeded with 17% EDTA and normal saline. After the root canal was dried with sterile paper tips, the lateral condensation method was used to obturate with gutta‐percha and AH Plus sealer (Dentsply Sirona, Ballaigues, Switzerland). Finally, the tooth was temporarily restored, followed by confirmation radiography, and referred for final restoration (Figure 3c).

## 4 Conclusion and Results

**FIGURE 4:**
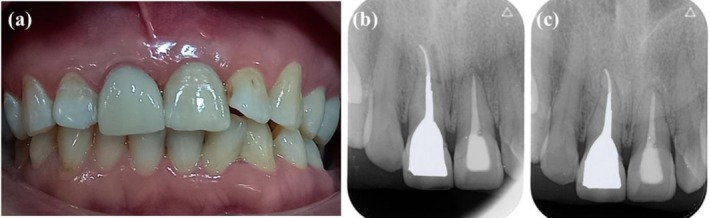
(a) Postoperative clinical photography of a permanent restoration. (b) 1‐month follow‐up radiograph after permanent restoration. (c) 12‐months follow‐up radiograph showing periapical healing.

After a 1‐month follow‐up, the tooth was permanently restored and the patient had not reported any symptoms since the treatment. Also, the tooth was not sensitive to percussion (Figure 4a,b). Twelve months following treatment, the patient attended a recall appointment. Clinical examination was performed, and a follow‐up periapical radiograph was taken to assess the tooth's healing progress. The tooth was symptom‐free, and the radiograph showed that the apical area had healed successfully (Figure 4c).

In this patient, the fiber post was removed swiftly and safely, reducing the amount of tooth structure lost and making the procedure less stressful for the patient and the practitioner. Guided endodontics is a suitable choice for the removal of fiber posts.

## 5 Discussion

The present case report details a guided method that uses CBCT and a 3D printer to remove the fiber post during nonsurgical endodontic retreatment. Since apical surgery was not an option due to the requirement of intracanal retention, a guided approach to tooth retreatment was proposed.

Fiber posts have been removed using various methods, but their removal presents significant challenges for clinicians. Overall, various methods can be employed to remove fiber posts, including ultrasonic vibrations, long‐shank burs, and specialized post removal kits [22]. Nevertheless, despite the presentation of dental operative microscopes and various methods to remove fiber posts, there are still risks such as axis deviation, root perforation, dentin loss, and eventual root weakness [23, 24]. Several factors involving post type, design, material, length, and cementing material make post removal difficult.

The fiber post can be precisely removed with the guided endodontic technique, which is reliable. According to the research results, endodontic guides successfully managed infected root canals with fiber posts [25, 26]. The study investigated the removal of fiber posts in the apical third of root canals with conventional and guided methods. The findings demonstrate that the guided method decreased dentin loss compared to the conventional technique [27]. In addition, the guided methods provide less angular deviation and volume loss compared to the microscope and ultrasonic techniques for fiber post removal in premolar and molar teeth [28, 29]. Some ex vivo studies conducted to assess the accuracy of guided endodontic [30, 31, 32] showed a low error in the deviation of the drill tip and angle deviation. The discrepancy may be greater since the drill must penetrate deeper into the root to remove the cemented post [33]. Furthermore, clinical conditions differ from those in ex vivo studies regarding the use of different CBCT settings, optical scanning methods, and implant planning software [34, 35]. Thus, more research is required to enhance the accuracy of guided endodontic, depending on several aspects.

CBCT is a noninvasive and reliable tool frequently used to diagnose and treatment plan for dentoalveolar disorders [36]. If careful analysis of periapical radiographs taken at different angles did not provide definitive information or if more information in the buccolingual dimension is still needed, a CBCT scan may be considered. In cases where a scan is necessary, a limited field of view is recommended due to its higher spatial resolution and lower radiation dose [36]. To assess teeth with suspected complex morphology, locate obliterated canals, plan nonsurgical and surgical endodontic retreatment, dentoalveolar trauma, evaluate the outcome of endodontic treatment, as well as root resorption, CBCT should only be employed as a complementary tool in specific clinical situations [37]. For instance, the guided endodontic method needs CBCT imaging, which entails more doses of radiation than periapical radiography. However, the American Association of Endodontists states that CBCT should only be utilized when a clinical examination and the patient's history show that the patient's advantages outweigh the potential risks. The present case serves as an indication for the application of CBCT [36].

Various digital planning software is employed to fabricate the endodontic guide. The reduction of deviations is achieved through precise planning and printing. Different digital planning software can result in varying degrees of guide design accuracy. The merging technique of DICOM and STL files can also influence the guide's accuracy [38]. The automatic combination of the two files can result in a decrease in the precision of the guide. Artificial intelligence can be helpful for CBCT images segmentation and integration with STL models [39]. The thickness of the layers in the STL file can affect the accuracy of the printed guide. A printed guide with a thinner layer thickness is more reliable [40]. Light‐cured resin is commonly used in printing, and accurate curing is necessary to preserve the guide's dimensional stability. It's essential to adapt printer settings to the manufacturer's recommendations [41].

According to reports, guided endodontic is a clinically valuable and safe technique for less experienced clinicians [13]. Whereas, it is reported that there is a notable distinction between experienced and less experienced clinicians for fiber posts removal in mandibular molars. Accuracy may be impacted by an eccentric force applied by the less experienced clinician within the tolerance gap between the drill and guiding hole [42].

The tooth presented with both a cemented intracanal fiber post and a large periapical lesion, where nonsurgical retreatment was the only viable option due to maintaining the fiber post space, which was essential for the intracanal retention and the tooth's restoration. This situation differs from previous reports, which often describe post‐removal in teeth without significant periapical pathology. Additionally, most studies on fiber post removal have focused on the premolars and molars, with limited research involving anterior teeth having a remaining crown, where correct recognition of the drilling direction can be more challenging due to the greater inclination angle of the tooth axis in maxillary anterior teeth [35, 43]. Removing a fiber post from an anterior maxillary tooth while preserving dentin structure was particularly challenging, as conventional techniques would increase the risk of root perforation and structural weakening. The guided approach allowed precise and conservative removal, minimizing tooth structure loss. While most existing literature focuses on laboratory or ex vivo studies, this case adds clinical evidence demonstrating the safety and predictability of guided endodontics in managing complex retreatments involving intracanal fiber posts and periapical pathology, with successful healing confirmed at 12‐month follow‐up. Long‐term follow‐ups are necessary in teeth with periapical lesions. The majority of periapical lesions heal within 1 year; healing may continue for up to 4 years or longer [44]. However, in this case, as in the results of similar studies [23, 25, 34], periapical lesion healing was achieved during the 12‐month follow‐up period.

The guided approach described in this case report presents several limitations. First, the technique requires specialized clinician training and a learning curve. In addition, guided endodontic procedures rely on CBCT imaging to allow three‐dimensional evaluation of the target area. However, CBCT exposes patients to higher ionizing radiation levels than conventional radiographs, which may raise concerns for some individuals. Furthermore, the accuracy of the approach is highly dependent on the quality of intraoral scanning, virtual 3D planning, and guide fabrication, making it sensitive to potential distortions or errors at any stage. Another drawback is that guided endodontics allows only linear access, which restricts its applicability to straight canals, and the guide may lack stability in partially edentulous patients. Moreover, the guides' fixed angulation, size, and depth are not easily adjustable once fabricated. Finally, the costs associated with production and the time required for planning and manufacturing can pose practical challenges [16, 45, 46].

The rapid advancement of digital dental processes, aided by developing technology, will continue to enhance the accuracy of guided endodontics. This digitally assisted method's widespread application in dentistry will result from these improvements.

## Author Contributions

**Mamak Adel:** project administration, resources, validation, visualization, writing – review and editing. **Zohreh Asgari:** conceptualization, data curation, investigation, methodology, resources, writing – original draft.

## Ethics Statement

This case report meets the ethical guidelines and adheres to Iran's local legal requirements.

## Consent

Written informed consent was obtained from the patient to publish this report in accordance with the journal's patient consent policy.

## Conflicts of Interest

The authors declare no conflicts of interest.

## Data Availability

The data that support the findings of this study are available on request from the corresponding author. The data are not publicly available due to privacy or ethical restrictions.
